# Water Extract of *Chrysanthemum indicum* L. Flower Inhibits Capsaicin-Induced Systemic Low-Grade Inflammation by Modulating Gut Microbiota and Short-Chain Fatty Acids

**DOI:** 10.3390/nu15051069

**Published:** 2023-02-21

**Authors:** Bing Yang, Dongfang Sun, Lijun Sun, Yaokun Cheng, Chen Wang, Lianhua Hu, Zhijia Fang, Qi Deng, Jian Zhao

**Affiliations:** 1 College of Food Science and Technology, Guangdong Ocean University, Guangdong Provincial Key Laboratory of Aquatic Product Processing and Safety, Zhanjiang 524088, China; 2 College of Food Science and Technology, Nanjing Agricultural University, National Center of Meat Quality and Safety Control, Nanjing 210095, China; 3 School of Chemical Engineering, The University of New South Wales, Sydney, NSW 2052, Australia

**Keywords:** systemic low-grade inflammation, capsaicin, *Chrysanthemum indicum* L., gut microbiota, short-chain fatty acid

## Abstract

Systemic low-grade inflammation induced by unhealthy diet has become a common health concern as it contributes to immune imbalance and induces chronic diseases, yet effective preventions and interventions are currently unavailable. The *Chrysanthemum indicum* L. flower (CIF) is a common herb with a strong anti-inflammatory effect in drug-induced models, based on the theory of “medicine and food homology”. However, its effects and mechanisms in reducing food-induced systemic low-grade inflammation (FSLI) remain unclear. This study showed that CIF can reduce FSLI and represents a new strategy to intervene in chronic inflammatory diseases. In this study, we administered capsaicin to mice by gavage to establish a FSLI model. Then, three doses of CIF (7, 14, 28 g·kg^−1^·day^−1^) were tested as the intervention. Capsaicin was found to increase serum TNF-α levels, demonstrating a successful model induction. After a high dose of CIF intervention, serum levels of TNF-α and LPS were reduced by 62.8% and 77.44%. In addition, CIF increased the α diversity and number of OTUs in the gut microbiota, restored the abundance of *Lactobacillus* and increased the total content of SCFAs in the feces. In summary, CIF inhibits FSLI by modulating the gut microbiota, increasing SCFAs levels and inhibiting excessive LPS translocation into the blood. Our findings provided a theoretical support for using CIF in FSLI intervention.

## 1. Introduction

Certain foods, such as chili, litchi, pepper, etc., are known as “heating” foods in traditional Chinese medicine. The excessive consumption of “heating” foods may cause a number of disorders, such as red and swollen eyes, acne, sores and ulcers in the mouth and tongue, swollen gums, sore throat, yellow urine, constipation and other symptoms; these symptoms are known as “shanghuo” (heating-up) in Chinese medicine [1,2,3]. Modern medicine defines “shanghuo“ as a kind of systemic and chronic low-grade inflammation characterized by a significant increase in inflammatory factors such as tumor necrosis factor-α (TNF-α) [4]. Prolonged systemic low-grade inflammation can cause substantial damage to the body and induce chronic diseases such as obesity, diabetes and depression [5,6,7]. The mechanism of food-induced systemic low-grade inflammation (FSLI) has yet to be established. Generally, nonsteroidal anti-inflammatory drugs (NSAIDs) are used to treat high-grade inflammation clinically. However, FSLI is a long-term condition that requires prolonged periods of medication, but such long-term treatment with NSAIDs can induce a number of side effects in the gastrointestinal tract, liver, nervous system and other organs of the body [8]. Therefore, persistent FSLI requires more appropriate prevention and intervention strategies with fewer side effects. Traditional Chinese medicine believes that medicine and food have a homologous relationship, and food components can also act as medicine to prevent and treat various disorders. Thus, according to this tradition, some foods are commonly used to replace anti-inflammatory drugs to inhibit inflammatory “shanghuo”. The *Chrysanthemum indicum* L. flower (CIF) is one such herb that is often added to tea to relieve sore throats. Several reports have shown that CIF has antioxidant activity in vitro [9,10,11,12]. However, the mechanism of action of CIF in FSLI has yet to be elucidated to provide a solid scientific foundation for its applications as a herbal medicine in preventing and treating FSLI.

Previous studies have shown that FSLI is associated with a diet-induced disorder in the intestinal microbiota [13,14]. However, in most of the existing studies, the direct injection of LPS was used to establish the inflammatory model, which may not represent the situation of low-grade inflammation induced by unhealthy diet. To overcome this problem, a food-induced model of systemic low-grade inflammation needs to be established. Studies have reported that the excessive consumption of chili in some people can induce inflammatory “shanghuo” [1,15,16]. However, the role of gut microbiota in the process of inflammation induced by chili has not been reported in the literature.

In this study, capsaicin, a major component of chili (a representative “heating” food), was used to establish a FSLI model by the oral gavage of mice for 7 days. The CIF were then orally administered to the mice at different doses as the treatment. Inflammatory factors, gut microbiota and SCFAs were analyzed to determine their correlations. The objective of the study was to investigate the anti-inflammatory effect of CIF on capsaicin-induced FSLI and elucidate its mechanism of action, so as to provide a theoretical basis for the development of functional foods and nutritional supplements with efficient anti-systemic inflammation effects.

## 2. Materials and Methods

### 2.1. Preparation of CIF Extract

Dry CIF were purchased from Antai Biotechnology Co., Ltd., (Shenzhen, China). They were mixed with distilled water in 1:8 ratio (*w*/*w*), heated and simmered for 1 h for three sessions, followed by filtration with busher funnel. The filtrate was concentrated to 400 mL at (47 ± 1 °C) by rotary evaporator (N-1100V-WB, Tokyo Physicochemical Equipment Co., Tokyo, Japan). The concentrate was desiccated in a laboratory oven for 62 h at 60 °C to produce the CIF powder. The CIF powder was stored at −80 °C in a refrigerator and diluted to 0.4, 0.2, 0.1 g/mL with pure water before use. The main components of CIF extract were 3,4-dihydroxybenzoic acid, chlorogenic acid, luteoloside and linarin. The extraction methods and the main components are reported in the literature [17,18].

### 2.2. Preparation of Laboratory Animals

Six-week-old female C57BL/6 J mice, each weighing 20 ± 2 g, were purchased from Beijing HuaFukang Biotechnology Co., Ltd., (Beijing, China) and raised in specific pathogen-free (SPF) conditions, with the license number SCXK (Beijing) 2014007. The mice were first acclimated to an environment of 25 ± 2 °C, humidity 55% ± 5% and a 12 h light/dark cycle. During the experiment, adequate feed and water were provided. After 3 days of adaptive feeding, the mice were randomly divided into 5 treatment groups: model group (CHCB group), low-dose group (CHL group), medium-dose group (CHM group) and high-dose group (CHH group), along with a blank control group (CHCA group). Mice in treatment groups were treated with 14.4 g·kg^−1^·day^−1^ capsaicin (purity > 99%, Guangzhou Bosen Pharmaceutical Co., Ltd., Guangzhou, China) solution orally for 7 days. Starting from day 10, mice in the CHL, CHM and CHH groups were treated with 7, 14 and 28 g·kg^−1^·day^−1^ of CIF for 3 days. The lowest dose used in this study was 567 mg/kg as human dose, which was within the range of 278.17 ± 358.0 mg/kg reported in the literature [19]. To convert human dose to mice dose, multiply human dose by 12.3 to get 7 g·kg^−1^ according to the literature [20]. The medium and high doses were 2 times and 4 times those of low doses, respectively.

### 2.3. Determination of LPS and Inflammatory Factors in the Serum of Mice

At the end of treatment, the mouse blood was collected into a pyrogen free centrifuge tube. The blood was maintained at 25 °C for 30 min and centrifuged in a refrigerated centrifuge at 8000 rpm and 4 °C for 5 min and the serum was collected. Lipopolysaccharide (LPS) in the serum was assessed using the limulus amebocyte lysate kit (Xiamen Limulus Reagent Biotechnology Co., Ltd., Xiamen, China). Serum levels of tumor necrosis factor-α (TNF-α), interleukin-1β (IL-1β), interleukin-6 (IL-6) and interleukin-10 (IL-10) were determined by respective ELISA kits (Shenzhen Xinbosheng Biotechnology Co., LTD., Shenzhen, China).

### 2.4. Analysis of Mouse Gut Microbiota

Mice feces were directly collected using a 1.5 mL sterile centrifuge tube on the last day of the experiment. Fecal samples were analyzed by 16SrDNA high-throughput sequencing method. Then, sample species information was obtained by comparing with database to obtain species category of gut bacteria. The methods of analyzing gut microbiota followed the methods described in [21].

### 2.5. Determination of SCFAs Content

A 50 mg fecal sample (collected in 2.4) was added to 100 μL of 15% phosphoric acid, 100 μL of 125 μg/mL isohexic acid solution as internal standard and 900 μL of ether and the mixture was homogenated for 1 min. The suspension was centrifuged at 12,000 RPM and 4 °C for 10 min and the supernatant was filtered through a 0.22 μm organic micropore membrane. Acetic acid, propionic acid, butyric acid, isobutyric acid, valeric acid and isovaleric acid in the filtrate were analyzed by gas chromatography mass spectrometry (GDC/GC-MS-QP2010, Shimadsu, Japan). A total of 1 μL of sample was injected into GC-MS, which was equipped with a VF-Wax column. Helium was the carrier gas at a flow rate of 1.0 mL/min. The injection temperature was 250 °C and the ion source temperature was 230 °C.

### 2.6. Statistical Analysis

SPSS 24.0 was used to analyze all the results, and each analysis was replicated 5 times (n = 5). All experimental data were expressed as mean ± SD. One-way Analysis of Variance (ANOVA) was performed to compare means in different groups, and a value of *p* < 0.05 was considered statistically significant.

## 3. Results

### 3.1. Effects of Capsaicin and CIF on the Levels of Serum Inflammatory Factor in Mice

**Figure 1:**
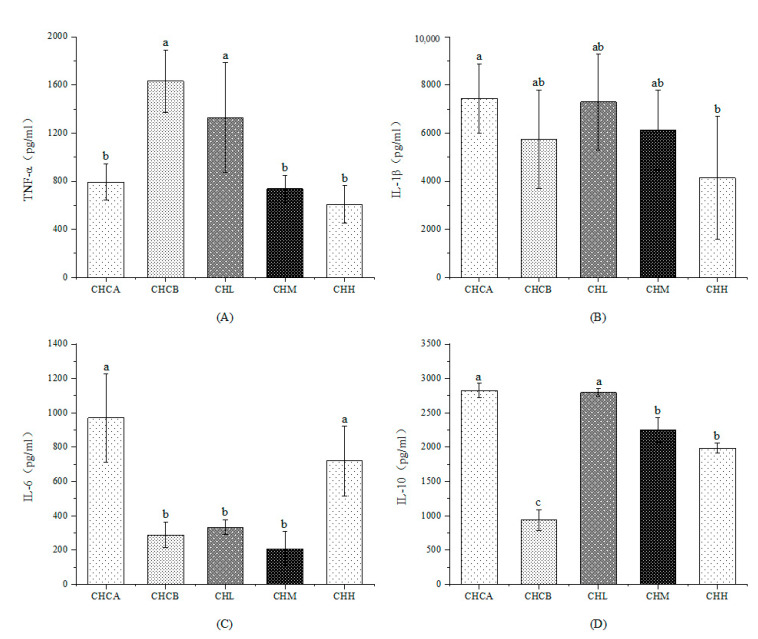
Effects of CIF on serum inflammatory factors level. (**A**): TNF-α, (**B**): IL-1β, (**C**): IL-6, (**D**): IL-10. Values indicate mean ± SEM (n = 5). a, b, c Values not sharing a common letter are significantly different among the groups (*p* ≤ 0.05).

To determine whether capsaicin treatment can induce inflammation in mice and the effects of CIF on capsaicin-induced inflammation, the typical inflammatory factors TNF-α, IL-1β, IL-6 and IL-10 in the mouse serum were measured (Figure 1). Compared with the CHCA group, the serum TNF-α level in the CHCB group was increased by 105.8% (*p* < 0.05), while IL-6 and IL-10 were decreased by 70.4% and 30.4% (*p* < 0.05), respectively. These results demonstrated that capsaicin gavage successfully induced FSLI in the mice. On the other hand, compared with the CHCB group, the TNF-α level of mice in the CHM and CHH groups decreased by 54.9% and 62.8% (*p* < 0.05), respectively. Furthermore, treatment with high-dose CIF increased the levels of IL-6 in the blood of mice by 150.4% (*p* < 0.05), while the levels of IL-10 in serum significantly increased (*p* < 0.05) in all groups. In sum, CIF administration led to decreases in the proinflammation factors (TNF-α) and increases in the level of anti-inflammation factors (IL-6 and IL-10) in the serum of FSLI mice.

### 3.2. Effects of Capsaicin and CIF on Serum LPS Levels in Mice

**Figure 2:**
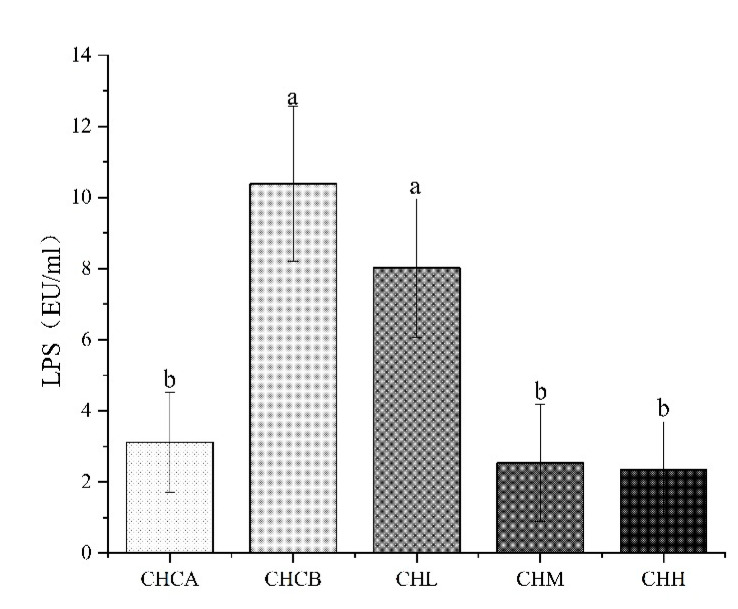
Effects of Water extract of CIF on serum LPS level. Values indicate mean ± SEM (n = 5). a, b. Values not sharing a common letter are significantly different among the groups (*p* ≤ 0.05).

Lipopolysaccharide(LPS) is a major component of the cell wall of gram-negative bacteria, and can induce inflammation in vivo when circulating in the blood of the organism [22]. The LPS in the mouse serum was measured in this study (Figure 2). Compared with the CHCA group, LPS concentration in the serum of mice in the CHCB group increased 2.33-fold (*p* < 0.05). After intervention with medium and high doses of CIF, the LPS levels in the treatment groups decreased by 75.56% and 77.44%, respectively (*p* < 0.05). These results further confirmed that capsaicin gavage induced FSLI in the mice, and treatment with medium and high doses of CIF effectively reduced LPS in the FSLI mice.

### 3.3. Effects of Capsaicin and CIF on the Diversity of Intestinal Microflora in Mice

Alpha (α) diversity is one of the important indices reflecting the abundance, evenness and diversity of gut microbiota. As shown in Table 1, the Shannon, Simpson, Chao1 and Ace indices of the CHCB group were significantly lower (*p* < 0.05) compared with the CHCA group, demonstrating that feeding mice with capsaicin significantly reduced the abundance and diversity of gut microbiota in the mice. On the other hand, all of the indices in the CHL and CHH groups were significantly higher than in the CHCB group (*p* < 0.05), suggesting that the intervention with low and high doses of CIF enhanced and restored the richness and diversity of gut microbiota that had been affected by feeding the mice with capsaicin.

**Figure 3:**
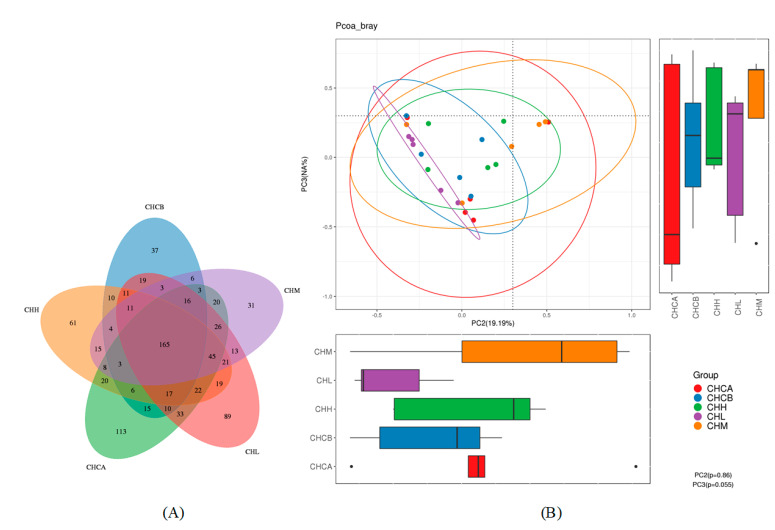
Effects of capsaicin and CIF on the richness and diversity of the gut microbiota. (**A**): Venn diagram of the number of OTUs of intestinal contents between experimental and control groups. (**B**): principal coordinate analysis (PCoA) of intestinal flora structure of mice in experimental and control groups.

The Venn diagram presents a breakdown of the common and unique operational taxonomic unit (OTU) numbers among each group (Figure 3). Compared with the 287 OTUs in the CHCA group, a markedly reduced number of 101 OTUs were found to be unique to the CHCB group. The number of OTUs endemic to the CHCB group was 37, which was considerably lower than the 89 OTUs endemic to the CHL group and 68 OTUs endemic to the CHH group (Figure 3A). The distances between the samples were determined in the principal coordinate analysis (PCoA), which reflects the difference in β diversity of gut microbiota (Figure 3B). The results were not significant, suggesting similar structural characteristics of gut microbiota in each group. In sum, the feeding of mice with capsaicin reduced the richness and diversity of their gut microbiota, while treatment with CIF restored and enhanced the richness and diversity.

### 3.4. Effects of Capsaicin and CIF on Gut Microbiota at Phylum and Genus Levels in Mice

**Figure 4:**
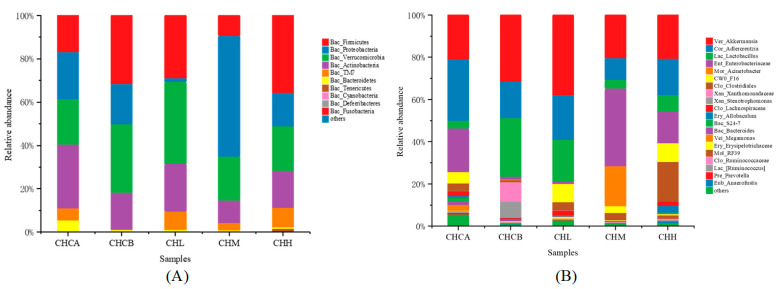
Effects of capsaicin and CIF on the composition and proportion of gut microbiota in mice. (**A**): At the phylum level, (**B**): At the genus level.

Figure 4 shows the effects of capsaicin and CIF on the gut microbiota composition of mice. At the phylum level, the abundance of Bacteroidetes and TM7 in the CHCB group decreased by 79.63% and 97.99%, respectively, while the abundance of Firmicutes increased by 86.15% (*p* < 0.05) compared with the CHCA group, suggesting that the structure of the gut microbiota was significantly altered by feeding the mice with capsaicin. After CIF intervention, however, the abundance of TM7 was significantly increased 7.73-, 2.90- and 7.90-fold in the CHL, CHM and CHH groups, respectively, compared with the CHCB group, while the abundance of Bacteroidetes did not change significantly (*p* < 0.05) (Figure 4A). These results suggest that the CIF restored the structural changes in gut microbiota caused by capsaicin.

At the genus level, feeding the mice with capsaicin caused the abundance of *Xanthomonas*, *Stenotrophomonas*, *Anaerofustis* and *Lactobacillus* in the CHCB group to increase 340.87-, 730.97-, 6.38- and 6.49-fold (*p* < 0.05), while the abundance of *Ruminococcus* was decreased by 65.5%, compared with the CHCA group. However, CIF intervention resulted in the levels of *Xanthomonas* and *Stenotrophomonas* in inflamed mice decreasing by more than 99.7% compared with the CHCB group. The abundance of *Anaerofustis* in the CHL and CHM groups decreased by 76.62% and 83.25% (*p* < 0.05), respectively, compared with the CHCB group. The levels of *Lactobacillus* in the CHL, CHM and CHH groups decreased by 28.57%, 84.94% and 71.40%, respectively (*p* < 0.05), while the abundance of *Ruminococcus* species increased by 137.74%, 61.50% and 263.90%, respectively, compared with the CHCB group.

In sum, feeding the mice with capsaicin caused a significant decrease in the species diversity of their gut microbiota, and changes in the dominant species, while CIF treatment restored the species diversity reduced by capsaicin.

### 3.5. Correlation between Inflammatory Factors and Gut Microbiota

**Figure 5:**
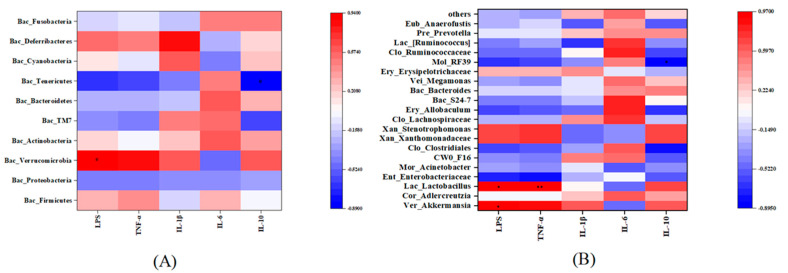
Heatmap of correlations between inflammatory factors and gut microbiota. (**A**): Correlation between inflammatory factors and gut microbiota at the phylum level. (**B**): Correlation between inflammatory factors and gut microbiota at the genus level. The color of the block represents the correlation between inflammatory factors and bacteria. * represents a significant correlation (*p* < 0.05); ** represents most significant correlation (*p* < 0.01).

To find the relationship between capsaicin-induced inflammation and the gut microbiota in the mice as well as the mechanism of the CIF treatment, the Pearson correlation coefficient was used to analyze the correlation between inflammatory factors and gut microbiota (Figure 5). The change in the proportion of *Verrucomicrobia* was positively correlated with LPS. There was a negative correlation between *Tenericutes* and IL−10 level. The number of *Lactobacillus* had a significant positive correlation with LPS and TNF−α (*p* < 0.05), while the number of *Akkermansia* was correlated with the level of LPS (*p* < 0.05). In addition, RF−39 was negatively correlated with the level of IL−10 (*p* < 0.05).

### 3.6. Effects of Capsaicin and CIF on SCFAs Content of Feces in Mice

**Figure 6:**
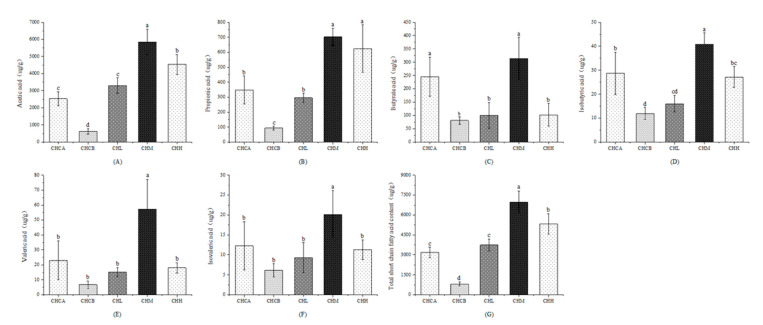
Effects of capsaicin and CIF on the SCFAs of feces. (**A**): Acetic acid, (**B**): Propionic acid, (**C**): Butyric acid, (**D**): Isobutyric acid, (**E**): Valeric acid, (**F**): Isovaleric acid, (**G**): Total short-chain fatty acids. Values indicate mean ± SEM (n = 5). a, b, c, d Values not sharing a common letter are significantly different among the groups (*p* ≤ 0.05).

Feeding the mice with capsaicin led to significant reductions in the level of SCFAs in the feces of the mice (Figure 6). The levels of acetic acid, propionic acid, butyric acid, isobutyric acid and total SCFAs in the CHCB group were reduced (*p* > 0.05) by 75.5%, 73.1%, 67.0%, 58.5% and 74.3%, respectively, compared with those in the CHCA group, while the concentrations of valeric acid and isovaleric acid did not differ significantly. On the other hand, the treatment of the capsaicin-fed mice with CIF resulted in restoration and increases in the SCFA levels in the mice. Compared with the CHCB group, the total SCFA production in the CHL, CHM and CHH groups increased by 357.0%, 751.6% and 549.5%, respectively (*p* < 0.05), while butyric acid in these groups increased (*p* < 0.05) by 23.1%, 287.3% and 25.9%, respectively. Total SCFAs and acetic, propionic, isobutyric, isovaleric and valeric acids were increased (*p* < 0.05) by 843.1%, 650.2%, 242.0%, 227.9% and 746.6% in the CHM group, respectively, compared with the CHCB group. The levels of these acids in the CHH group increased by 632.5%, 565.7%, 127.7%,84.5% and 167.5%, respectively, compared with the CHCB group. Overall, these results indicate that capsaicin intervention significantly reduced the production of SCFAs in mice, while treatment with CIF significantly increased the production of SCFAs. Medium and high doses of CIF effectively increased the synthesis of SCFAs in mice.

## 4. Discussion

Previous studies use direct LPS injection to induce systemic low-grade inflammation in laboratory animals such as mice [23]. The drawback of this practice is that the inflammatory effects induced by LPS may not be the same as or accurately represent those induced by unhealthy diet. To overcome this drawback, an inflammation model induced by food components is needed. Capsaicin is the component in chili that gives the “hot” sensation and is widely believed in Chinese medicine to cause “shanghuo” or heating up symptoms in the body. Some studies have reported the beneficial effects of low-dose capsaicin on the health of mice [24,25,26], while others have shown that high doses of capsaicin can cause intestinal inflammation and physiological disorders [27,28,29,30]. In the present study, a FSLI model via the oral administration of high doses of capsaicin was established. Our results showed that high doses of oral capsaicin significantly increased the serum TNF-α levels in mice to almost twice those of the control group, indicating that excessive capsaicin can induce FSLI in mice. Our findings demonstrate the feasibility of establishing a model of FSLI using capsaicin, which was reported here for the first time.

*Chrysanthemum indicum* L. is a herb that is commonly added to tea or infused directly as a tea analogue in China to prevent or alleviate “hotting up” symptoms, especially in summer time. People usually add 25–50 g of CIF to 250–500 mL of water, so many studies use the water extract of CIF with concentrations in the range of 0.1–0.2 g/mL [31,32]. The lowest concentration in this study was 0.1 g/mL, which was in line with people’s habitual intake. As a Chinese herbal medicine, the average human equivalent dose value is 278.1 ± 358.0 mg/kg, and the values for single-herb are 322.7 ± 488.4 mg/kg [19]. The lowest dose used in this study was 567 mg/kg as a human dose, which was within the range reported in the literature. Medium and high doses were 2 times and 4 times those of low doses, respectively. *Chrysanthemum indicum* L. is rich in polyphenols such as luteolin, caryolane, acacetin, apigenin, which have been shown to have anti-inflammatory activity [33]. However, there is relatively scant information on the effect of CIF as a whole in treating systemic low-grade inflammation. In this study, we found that exposure to medium and high doses of CIF significantly reduced the levels of the inflammatory factor TNF-α, which was significantly increased by feeding the mice with capsaicin, implying that CIF was able to alleviate capsaicin-induced systemic inflammation. Also, we found that the serum IL-6 level decreased significantly in capsaicin-induced systemic inflammation, contrary to previous reports that cancer-related chronic inflammation is accompanied by an increase in IL-6 [34]. Treatment with high-dose CIF led to significantly elevated levels of IL-6, restoring it to the levels in the control group. Furthermore, feeding capsaicin to mice significantly reduced their level of serum IL-10. Previous reports have shown that IL-10 is an anti-inflammatory factor that inhibits the synthesis of pro-inflammatory factors [35]. The IL-10 levels in the mice significantly increased after the CIF intervention. Taken together, these results demonstrated that CIF was able to reduce systemic low-grade inflammation induced by capsaicin by lowering the proinflammation factors such as TNF-α and increasing the level of anti-inflammation factors including IL-6 and IL-10.

Previous studies have shown that excessive LPS translocation into the blood is one of the main causes of systemic inflammation [36,37]. However, it is not clear whether food-induced FSLI is mediated via a similar mechanism. The results obtained in this study showed that the concentration of LPS in the inflamed mice was more than twice that of the control group, suggesting that high-dose capsaicin promoted excessive LPS translocation into the blood and induced systemic inflammation. On the other hand, CIF treatments significantly decreased the levels of LPS in serum, indicating that CIF effectively prevented the release of a large amount of LPS into the blood, and thereby reduced the level of inflammation. This agreed with previous in vitro cell culture studies that showed that CIF treatments reduced LPS-induced inflammation. As the concentration of LPS in blood is implicated in intestinal mucosal permeability, it can be speculated that CIF might reduce the intestinal barrier permeability, thus preventing the release of LPS into the blood and reducing the level of inflammation. However, further studies are needed to confirm the effects of enhancing intestinal barrier integrity.

In the past 10 years, there have been numerous studies investigating the role of dietary intake in altering gut microbiota. However, these studies are focused on the effects of major food components such as fat, sugar and dietary fiber on the gut microbiota, while relatively few studies have examined the effect of minor components such as capsaicin on gut microbiota and their relationship with systemic inflammation, especially induced by unhealthy diet. An appropriate amount of capsaicin is found to have a positive regulatory effect on the structure and function of gut microbiota [25]. In the present study, high-dose capsaicin caused significant decreases in both the abundance and diversity of the gut microbiota of mice. Moreover, the structure of microflora also changed significantly. Gut microbiota have been reported to play a key role in the development of inflammation [38], and alterations of gut microbiota caused by capsaicin are a potential pathway for its inflammatory effects. On the other hand, intervention with medium and high doses of CIF restored the abundance and diversity of gut microbiota, indicating that CIF probably inhibited FSLI by modulating the structure of gut microbiota. One unanticipated finding was that the abundance of *Lactobacillus* increased significantly after the oral administration of high-dose capsaicin, while the Pearson correlation analysis indicated that the number of *Lactobacillus* were significantly related to the Serum LPS and TNF-α content. This result contradicted the common belief that an increased abundance of *Lactobacillus* can reduce inflammation levels [39,40,41], indicating that large increases in *Lactobacillus* may also contribute to systemic inflammation. Wang et al. found that CIF can decrease the abundance of *Lactobacillus* in metabolic hypertensive rats [42], but no studies have shown whether CIF can decease the abundance of *Lactobacillus* in a FSLI model. This study found that intervention with medium and high doses of CIF can decrease the amount of *Lactobacillus* increased by high-dose capsaicin, and the abundance of *Lactobacillus* was not significantly different with the control group, which partially agreed with our results. One of the main phenolic components of CIF, chlorogenic acid, has been reported to improve the relative abundance of *Lactobacillus* in a mouse model of dextran sulfate sodium-induced colitis [43]. However, the results of this study found that CIF decreased the abundance of *Lactobacillus*. The effect of CIF may be to restore the abnormal abundance of *Lactobacillus* induced by high-dose capsaicin, rather than simply affecting its rise or fall. Pearson correlation analysis indicated that the number of *Lactobacillus* were significantly related to the Serum LPS and TNF-α content, which is closely related to the level of FSLI. The large increase in *Lactobacillus* may potentially contribute to capsaicin-induced gut microbiota disorder and inflammation, but further studies are needed to confirm the hypothesis. After the intervention with medium and high doses of CIF, the abundance of *Lactobacillus* became comparable to that of the control group, indicating that CIF can effectively rebalance the structure of gut microbiota by restoring the abundance of *Lactobacillus*. The main components of CIF are polyphenols; most polyphenols (90–95%) cannot be absorbed by the gastrointestinal tract directly, but are transported to the colon and synthesized by specific bacteria to synthesize SCFAs [44].

Short-chain fatty acids play a key regulatory role in a variety of metabolic functions of the host. Butyric acid can adjust tight junction proteins to enhance the function of the intestinal barrier, while acetic acid and butyric acid inhibit intestinal inflammation [45,46,47]. Medium and high doses of CIF significantly increase the concentrations of acetic acid and butyric acid in mouse feces. Further, CIF treatment significantly increased the abundance of *Ruminococcaceae*, which are a family of butyrate-producing bacteria, suggesting that CIF promotes the synthesis of butyric acid, with the consequent enhancement of the intestinal barrier function, which in turn can hinder the entry of LPS into the blood and reduces the level of inflammation.

## 5. Conclusions

This study shows that excessive amounts of capsaicin can trigger gut microbiota disorder, increase the degree of LPS released into the blood and induce low-grade inflammation in the system. Therefore, capsaicin can be used to establish a FSLI model. On the other hand, treatments with medium and high doses of CIF can help restore the structure of gut microbiota, increase the production of SCFAs such as butyric acid, prevent the entry of LPS into the blood and inhibit FSLI. These findings provide scientific evidence to support the use of CIF for preventing and treating inflammation induced by unhealthy diet and are a foundation for the development of CIF-based functional foods and drinks for the prevention of chronic low-grade inflammation-related disorders.

## Figures and Tables

**Table 1:** The alpha diversity of intestinal microbiota in mice.

	Shannon	Simpson	Chao1	Ace	Goods_Coverage
CHCA	4.835 ± 0.409 ^a^	0.920 ± 0.017 ^a^	260.974 ± 48.874 ^ab^	253.707 ± 55.233 ^ab^	0.999
CHCB	1.949 ± 0.538 ^b^	0.554 ± 0.185 ^b^	141.000 ± 17.298 ^c^	144.3369 ± 17.075 ^c^	0.999
CHL	3.694 ± 0.548 ^ab^	0.851 ± 0.071 ^ab^	287.075 ± 22.114 ^a^	285.381 ± 19.954 ^a^	0.999
CHM	2.636 ± 1.474 ^b^	0.574 ± 0.256 ^b^	211.657 ± 35.323 ^b^	210.172 ± 35.070 ^bc^	0.999
CHH	3.578 ± 0.687 ^ab^	0.805 ± 0.086 ^ab^	244.786 ± 19.736 ^ab^	238.95 ± 21.048 ^ab^	0.999

Values indicate mean ± SEM (n = 5). a, b, c Values not sharing a common letter are significantly different among the groups (*p* ≤ 0.05).

## Data Availability

Sequencing reads were deposited in the NCBI’s sequence read archive under accession number PRJNA906648. Further data that support the findings of this study are available from the corresponding author upon reasonable request.

## Abbreviations

**Abbreviations**	**Full Name**
CIF	*Chrysanthemum indicum* L. flower
FSLI	food-induced systemic low-grade inflammation
IL-10	Interleukin-10
IL-1β	Interleukin-1beta
IL-6	Interleukin-6
LPS	lipopolysaccharide
NSAIDs	nonsteroidal anti-inflammatory drugs
OTU	operational taxonomic unit
PCoA	principal coordinate analysis
SCFAs	short-chain fatty acids
TNF-α	tumor necrosis factor-α
